# Negative Trauma Appraisals and PTSD Symptoms in Sri Lankan Adolescents

**DOI:** 10.1007/s10802-015-9985-y

**Published:** 2015-02-19

**Authors:** Thyagi Ponnamperuma, Nancy A. Nicolson

**Affiliations:** 1 Department of Community Medicine, Faculty of Medicine, University of Ruhuna, Matara, Sri Lanka; 2 Department of Psychiatry & Psychology, School for Mental Health and Neuroscience, Maastricht University, Box 616, 6200 MD Maastricht, The Netherlands

**Keywords:** PTSD, Negative appraisals, Cognitive model, Adolescents, Disaster, Internalizing symptoms

## Abstract

The cognitive model posits that negative appraisals play an important role in posttraumatic stress disorder, in children as well as in adults. This study examined correlates of negative appraisals in relation to trauma exposure and their relationship to posttraumatic stress symptoms (PTSS) in 414 Sri Lankan adolescents, aged 12 to 16, living in areas impacted in varying degrees by the 2004 tsunami. In 2008, participants completed measures of negative appraisals, lifetime traumatic events, posttraumatic stress symptoms, internalizing symptoms, ongoing adversity, and social support. The majority (70 %) of the participants reported multiple traumatic events; 25 % met DSM-IV criteria for full or partial PTSD. Adolescents who had experienced more severe events, abusive events, greater cumulative trauma, or greater current adversity reported more negative appraisals. In regression analyses controlling for known risk factors such as female gender, cumulative trauma, ongoing adversity, and low social support, negative appraisals were the best predictor of PTSS, explaining 22 % of the variance. This relationship appeared specific to PTSS, as negative appraisals did not predict internalizing symptoms. Findings confirm the link between negative cognitions concerning traumatic events and persistent PTSS in adolescents, but longitudinal studies are needed to determine whether appraisals contribute to symptom maintenance over time.

Traumatic experiences are common in the general population, but only a minority of exposed individuals develops trauma-related psychopathologies such as posttraumatic stress disorder (PTSD; Kessler et al. 1995). Of those who do develop PTSD, many recover within a few months, whereas others experience debilitating symptoms for years. The cognitive model, as formulated by (Ehlers and Clark 2000), posits that PTSD symptoms arise and are maintained when an individual processes the traumatic event in a way that leads to sense of current threat and subsequent anxiety. Maladaptive behavior and cognitive strategies result in temporary relief, but this prevents changes in negative appraisals and memory, thereby leading to persistent symptoms. Research, including a number of studies with prospective, longitudinal designs (Dunmore et al. 2001; Ehring et al. 2006), has provided empirical support for this model in adults. Results have demonstrated that negative appraisals of the trauma and its sequelae independently predicted long-term PTSD, over and above the initial symptom level.

The cognitive model has been extended from adults to children and adolescents, taking possible developmental changes in how children encode and resolve trauma memories into consideration (Dalgleish et al. 2005; Meiser-Stedman 2002; Salmon and Bryant 2002). Similarly to adults, children may interpret traumatic experiences and subsequent symptoms (for example, intrusive thoughts and images) as evidence of personal weakness or permanent damage, and of the world as a dangerous place. Such maladaptive cognitions maintain a sense of ongoing threat and can thus lead to persistent PTSD symptoms, long after the traumatic event. Understanding the process by which negative appraisals arise and exacerbate PTSD symptoms is also key to designing effective interventions in children and adolescents. Previous research has shown that cognitive behavioral therapy leads to clinical improvement in childhood PTSD primarily through changing maladaptive appraisals (Dalgleish et al. 2005; Smith et al. 2007).

Surprisingly little appears to be known about individual characteristics or features of traumatic experiences that increase the probability that a trauma-exposed child will develop negative cognitions concerning self and the world. Demographic characteristics such as gender and age, for example, might be relevant. In one study of children aged 7–13 years, younger children were more likely than older children to display maladaptive appraisals, as measured with the Children’s Posttraumatic Cognitions Inventory (CPTCI), 1–4 weeks after hospital admission for a traumatic injury (Salmon et al. 2007). In contrast, a validation study found no age differences on CPTCI total or subscale scores in a larger (*n* = 563) sample aged 6-18 years (Meiser-Stedman et al. 2009b). Given that female adolescents show a greater tendency to ruminate in response to stressful events (Trickey et al. 2012), one might expect more negative cognitive appraisals in girls. However, it appears that few studies have specifically examined gender differences in appraisals in this age group. One study reported higher CPTCI total scores in girls compared to boys (Meiser-Stedman et al. 2009b), whereas other findings suggest that gender is only weakly associated with trauma appraisals (Stallard and Smith 2007). The relationship of trauma characteristics to negative appraisals is also largely unexplored. There is some evidence that more severe or life-threatening traumas are associated with more negative cognitions. For example, motor vehicle accident (MVA) victims who described their injuries as more severe displayed more negative appraisals (Tierens et al. 2012). Different types of traumatic experiences are thought to be more or less likely to give rise to negative appraisals, but here again there appear to be limited data. The vast majority of studies in children and adolescents have focused on single-incident trauma (most commonly motor vehicle and other accidents), with fewer investigations of longer-term exposures, for example to sexual or physical abuse. Events that affect large groups, like natural disasters or war, may be appraised differently than those affecting individuals; intentional acts of interpersonal violence may be interpreted more negatively than natural disasters or accidents (Bryant and Guthrie 2005; Meiser-Stedman et al. 2009b; Trickey et al. 2012). To our knowledge, only a handful of studies, in adults, have directly compared the effects of different types of trauma on either negative appraisals or psychopathological outcomes. In comparing accidents, disasters, assaults (both sexual and non-sexual), and life-threatening illness, a study among Chinese young adults found the highest PTCI total scores in assault victims (Su and Chen 2008). Grimm et al. (2012) compared adults’ responses to events ranging from terrorist attacks, floods, fire, and building collapse, concluding that sudden, violent events may lead to more negative cognitive processes than events (like floods) that victims had at least some time to prepare for. Post-trauma circumstances may also impact appraisal processes. Current stressors (poverty, poor housing, unsafe neighborhood, personal or parental health problems, familial disruption, etc.) are likely to heighten adolescents’ perceptions of the world as an unsafe place, especially if these conditions arose or worsened in the aftermath of the trauma (*secondary adversities*, Pfefferbaum et al. 2013). Social support, on the other hand, may be effective in reducing negative appraisals, as reported in studies of MVA victims (Tierens et al. 2012) and sexually abused adolescents (Bal et al. 2009).

The posited association between negative appraisals and posttraumatic stress symptoms has been documented in children and adolescents, in both acute stress disorder (Salmon et al. 2007) and established PTSD (Ehlers et al. 2003). However, the significance of appraisals in the development of PTSD can only be understood in relation to other known predictors. These include female gender, more severe trauma, an unfavorable social environment (low social support, poor family functioning), adverse living conditions, pre-existing or co-occurring mental health problems, and maladaptive coping strategies (Trickey et al. 2012). In addition, cumulative trauma has emerged as a powerful risk factor. Exposure to multiple traumatic events increases the risk of PTSD in children (Fairbank and Fairbank 2009), and in a large-scale epidemiological study among adults with PTSD, cumulative exposure has been associated with greater impairment, longer duration of illness, and higher comorbidity (Karam et al. 2014). These variables could potentially moderate the relationship between negative appraisals and PTSD symptoms, or their effects on symptoms might be mediated by negative appraisals. For example, effects of social support on children’s PTSD symptoms were partially mediated by negative appraisals in some studies (Bal et al. 2009; Tierens et al. 2012), but not in others (Ellis et al. 2009).

Given evidence that traumatic events can lead not only to PTSD but also to depression or other disorders, it is important to clarify how specific the association between negative trauma appraisals and symptoms is to PTSD. In adults, prospective studies on motor vehicle accident survivors showed that theory-derived cognitive models for PTSD and depression were, as hypothesized, fairly specific in their ability to predict the development of symptoms over time (Ehring et al. 2006; Ehring et al. 2008). Thus, negative appraisals of trauma and its aftermath were, among other cognitive variables derived from the Ehlers and Clark 2000 model of PTSD, better predictors of PTSD than were cognitive variables such as negative self-evaluation and perceived lack of social support, derived from theoretical models of depression. Findings of studies in younger samples exposed to various types of trauma suggest, however, that negative trauma appraisal is not a specific correlate or predictor of PTSS, but may also be associated to a greater or lesser extent with depressive or internalizing symptoms (Bal et al. 2009; Meiser-Stedman et al. 2009b; Spaccarelli 1995). Furthermore, studies in which PTSS is the only symptom outcome measure leave open the possibility that the apparent association between negative trauma appraisals and PTSS is an artifact of underlying depression (Foa et al. 1999).

In summary, despite evidence that appraisal processes play an important role in PTSD, several questions remain. In addition to gaps in our understanding of the effects of different types of trauma on appraisals and associations of appraisals with cumulative trauma exposure, current adversity, and internalizing symptoms, previous research on this topic seems to have been limited to the first 6–12 months post-trauma. Moreover, most previous studies were conducted in Western samples, raising questions about the generalizability of the findings. Cultures and religions may differ in how traumatic events are interpreted, and this will in turn influence individuals’ cognitive processing during and after the event (see, for example, Levy et al. 2009). Available data suggest that negative appraisal measures are reliable and valid in non-Western cultures (Su and Chen 2008); moreover, negative cognitions were found to predict PTSS in adult survivors of the 2004 tsunami in Sri Lanka (Lommen et al. 2009). However, sample sizes in these studies were relatively small. Given the widespread exposure to traumatic events among children and adolescents in the developing world (Fairbank and Fairbank 2009) and the need to develop effective and culturally sensitive interventions in this context (Bryant and Njenga 2006), larger studies of cognitive processes related to PTSD are needed.

The current study was therefore designed to clarify the role of negative cognitive appraisals in relation to PTSS in a large sample of adolescents exposed in varying degrees to a natural disaster (the 2004 tsunami, in southern Sri Lanka), as well as to other kinds of traumatic experiences. We focused on three central issues:

1. Predictors of negative appraisals. We explored the possible contributions of gender and age to individual differences in negative appraisal. We expected that negative appraisal scores would vary according to characteristics of the worst traumatic event, with events involving interpersonal violence and events experienced as more severe (i.e., meeting DSM-IV event Criterion A) being particularly likely to give rise to negative appraisals. Moreover, adolescents who had experienced the greatest cumulative trauma were expected to exhibit the most negative appraisals. Based on previous research, we expected that ongoing adversity (e.g., problems with parental employment, housing, school, health, etc.) would exacerbate negative trauma cognitions and that social support would buffer them. We further hypothesized that the effects of trauma characteristics on negative appraisals might be indirect, via their associations with PTSS.
2. Negative appraisals as predictors of PTSS. We hypothesized that adolescents with more negative appraisals in relation to their traumatic experiences would have more severe symptoms of PTSD, even after controlling for other potential risk factors, as listed above. In addition, we tested the extent to which negative appraisals may have mediated the effects of other risk factors on PTSS.
3. Specificity of the relationship between negative appraisals and PTSS. We hypothesized that negative trauma appraisals would be better predictors of PTSS than of internalizing symptoms. We also examined the possibility that the association between negative appraisals and PTSS might be secondary to underlying internalizing symptoms.

## Method

### Setting and Participants

The study was conducted in secondary schools in Galle District in southern Sri Lanka. Galle was hard-hit by the 2004 tsunami, with approximately 4,000 people killed and 35,000 displaced. We identified two semi-urban study locations similar in socioeconomic profile but differing in degree of tsunami exposure: Hikkaduwa area, in the severely affected coastal belt, and Bope-Poddala, 6 km inland. Government assessments documented that 9 of the 40 secondary schools in the Hikkaduwa area but none of the 18 schools in Bope-Poddala area had been directly affected by the tsunami. We selected five affected schools in Hikkaduwa and five unaffected schools in Bope-Poddala, such that selected schools in each area were representative of the three categories of secondary education in Sri Lanka. Study approval was obtained from the local ethics committee (Faculty of Medicine, University of Ruhuna), the Sri Lankan Ministry of Education, and the school principals. Informed consent was also obtained from the participants and their parents.

Data were collected between January and March 2008, approximately 3 years after the tsunami. All children in grades 8 and 10 present at school on the day of data collection were informed about study goals and methods. Fewer than 5 % of the children were absent, and fewer of those present 1 % declined. Participants completed the questionnaires in a separate classroom, as needed with the help of research assistants.

The initial sample included 755 participants: 53 % from the high tsunami impact area and 47 % from the lower tsunami impact area. Of these, 452 (60 % of the total) reported having experienced at least one lifetime traumatic experience. The sample for the current analysis comprised the 414 participants with one or more traumas and a non-missing total score on the negative appraisals scale.

### Measures

#### **Traumatic Events**

The adolescent version of the UCLA PTSD Reaction Index for DSM-IV^1^ (PTSD-RI; Steinberg et al. 2004) was used to assess lifetime exposure to 13 categories of traumatic events. We replaced the original category “being in a big earthquake that badly damaged the building you were in” with “being in the tsunami”. A cumulative trauma score was defined as the number of different event categories a participant endorsed. Participants reporting multiple trauma categories indicated which event was currently the most bothersome (Worst Event). The PTSD-RI assessed whether this worst event met DSM-IV PTSD Criterion A: at the time of its occurrence, the individual appraised the event as life threatening (A1, 7 items) and also reacted with extreme fear, horror, or disgust (A2, 6 items). We used Criterion A (dummy-coded 1 if the participant endorsed at least one A1 item and one A2 item; see Steinberg et al. 2004) to differentiate severe from milder trauma. We further characterized worst events as abusive (experiencing sexual or physical abuse, or witnessing domestic violence) or not.

#### **Posttraumatic Stress**

The PTSD-RI was also used to assess symptoms and diagnostic criteria. Of the 22 items, 20 are associated with DSM-IV-TR criteria B (re-experiencing), C (avoidance), or D (arousal). Participants rated how often they experienced each symptom during the past month on 4-point scales ranging from 0 *none of the time* to 4 *most of the time*. We generated a PTSD symptom severity score (PTSS) by summing scores on the 20 relevant items (Steinberg et al. 2004). When scores on either one (*n* = 50) or two (*n* = 13) items were missing, values were imputed as the mean of the remaining items. Based on PTSD-RI algorithms (Steinberg et al. 2004), individuals were categorized as having full PTSD (meeting DSM-IV criteria for all three symptom clusters), partial PTSD (meeting criteria for two of the three clusters), or no PTSD.

#### **Negative Appraisals**

Participants indicated, on 5-point scales ranging from 0 *not at all* to 4 *all the time*, how often during the last 4 weeks they had experienced negative thoughts in relation to their worst event. Eight items were derived from a 10-item trauma appraisal questionnaire developed for children (Stallard and Smith 2007): “I might go crazy”; “something must be wrong with me”; “this isn’t fair”; “I will never get over this”; “things are never going to be right again”; “I should have got over this by now”; “this has ruined my life”; “nowhere is safe”. Although the original scale differentiated four subscales, factor analysis of items chosen for the current study (all four from subscales Negative Interpretation of Symptoms and Permanent Change; one of three items from subscale Future Danger, and the single Injustice item) failed to reveal more than one subscale. We therefore used the total score as a measure of negative trauma-related cognitions (Cronbach’s alpha = 0.79).

#### **Ongoing Adversity**

This measure of chronic stress was adapted from the Long-term Difficulties Questionnaire (Rosmalen et al. 2012). Adolescents indicated, on 4-point scales (0 *no problems* to 3 *serious problems*), whether they had experienced problems within the last 4 weeks in each of the following areas: school, financial, living situation in the neighborhood, leisure activities, peer relations, family relations, contacts with other people, health, worries about family members, worries about the future. The 10 ratings were summed to obtain a total score.

#### **Social Support**

The Multidimensional Scale of Perceived Social Support (Zimet et al. 1988) measures perceived support from family members, friends, and a significant other. Sum scores for each 4-item subscale were calculated from ratings on 7-point scales (1 *strongly disagree* to 7 *strongly agree*). The MSPSS has been validated in adolescent samples with diverse ethnic backgrounds (Klineberg et al. 2006; Bruwer et al. 2008). Because children in the current sample found it difficult to define a significant other, we excluded the sum score for this Special Person subscale and used the total of Family and Friends support scores (Cronbach’s alpha = 0.76).

#### **Internalizing Symptoms**

Participants completed the 25-item adolescent self-report version of the Strengths and Difficulties Questionnaire (SDQ; Goodman 1999), previously translated into Sinhalese (www.sdqinfo.com). Items are rated on 3-point scales (0 *not true*, 1 *somewhat true*, 2 *certainly true*). Following recent recommendations (Goodman et al. 2010), we combined scores on the subscales Emotional Problems and Peer Problems to obtain a 10-item measure of internalizing symptoms (Cronbach’s alpha = 0.56).

### Statistical Analysis

Analyses were performed with SPSS, with two-tailed *p*-values ≤ 0.05 considered significant. To address the central hypotheses, we conducted three separate multiple regression analyses. The dependent variable (negative appraisal score, PTSS, or SDQ internalizing score) in each regression model was square root-transformed to normalize its distribution. Independent variables were entered into preliminary models; those with significant effects were retained in final models. To test hypotheses concerning mediation (e.g., the extent to which PTSS may have mediated effects of trauma characteristics on negative appraisals, or whether negative appraisal was a significant mediator of the effects of other variables on PTSS), we used the SPSS macro PROCESS (Hayes 2013a, 2013b), with bias-corrected bootstrap 95 % confidence intervals (1,000 samples) for testing indirect effects. Details of each analysis are presented under the relevant Results subsection.

## Results

### Sample Characteristics

The 414 participants included 225 girls and 189 boys, ranging in age from 12 to 16 years (mean 13.6). Because trauma exposure was an inclusion criterion for this analysis, adolescents attending schools in the high tsunami-impact area were overrepresented (*n* = 224, 64 %) compared to those from the less affected area (*n* = 150, 36 %).

### Traumatic Events

A majority (69.8 %) of the adolescents had been exposed to more than one type of traumatic event. On average, participants endorsed 2.8 different event types (range 1–10 of the 13 categories). Boys reported significantly more total trauma types than girls, with means of 3.1 vs. 2.6, *t* (412) = 2.95, *p* = 0.003. Cumulative trauma score was unrelated to age, *r*(414) = -0.06, *p* = 0.22.

Table 1 summarizes the types of traumatic events participants reported. In this sample, personal exposure to the tsunami was the most common of the 13 event categories, being reported by almost half of the adolescents. The tsunami was also most frequently named as the worst event and was the most common event meeting DSM-IV Criterion A. Altogether, however, exposure to the tsunami accounted for only 16 % of reported events and for fewer than one-third of all worst events and Criterion A events. Although 154 of the 358 participants who indicated when their worst event had occurred listed intervals ranging from 3 to 4 years ago, corresponding to the time of the tsunami disaster, 159 participants mentioned more recent dates (in the past 2 years), and another 45 participants reported events that had occurred 5 to 14 years before study enrollment.

**Table 1:** Types and frequencies of traumatic events

Trauma type	Events	% total events	% participants	Worst event	% participants^a^	Criterion A met	% participants^a^
Tsunami	187	16.0	45.2	123	30.3	105	25.7
Other disaster	73	6.2	17.6	23	5.6	15	3.6
Bad accident	114	9.7	27.5	31	7.6	20	4.9
Medical procedure	113	9.6	27.3	46	11.2	28	6.8
Sexual abuse^b^	11	0.9	2.7	4	1.0	3	0.7
Physical abuse at home^b^	83	7.1	20.0	16	3.9	15	3.7
Physical abuse outside home^b^	31	2.6	7.5	5	1.2	3	0.7
Saw violence at home^b^	94	8.0	22.7	26	6.4	15	3.7
Saw violence outside home	92	7.9	22.2	36	8.8	26	6.4
Saw a dead body	156	13.3	37.7	60	14.7	37	9.0
Violent death or injury of loved one	144	12.3	34.8	71	17.4	54	13.2
War	17	1.5	4.1	6	1.5	6	1.5
Other	56	4.8	13.5	13	3.2	11	2.7
Total	1,171	100.0	100.0	461	98.3	338	71.2

^a^ Because 5 of the 414 participants did not identify a “worst event”, *n* = 409 here. ^b^ Trauma types defined as “abusive event” in the analysis

### PTSD Symptom Severity and Diagnoses

PTSD symptomatology (PTSS) was unrelated to age, *r*(403) = -0.03, *p* = 0.59, but girls reported more severe symptoms than boys (*M*_girls_ = 18.8, *SD* = 13.5; *M*_boys_ = 145.3, *SD* = 12.8), *t* (401) = 2.61, *p* = 0.009. Diagnostic criteria for full PTSD were met by 36 of the 414 adolescents (9 %) in this trauma-exposed sample. An additional 66 adolescents (16 %) met criteria for partial PTSD. The prevalence of clinically significant PTSD in this sample was thus 25 %. Age showed no association with PTSD diagnostic categories. Although girls more often met PTSD symptom criteria than boys (girls: 10 % full, 18 % partial, 28 % combined; boys: 7 % full, 13 % partial, 21 % combined), this difference was not statistically significant (combined categories partial and full PTSD versus no PTSD: *Χ* ^2^ (1, *N* = 414) = 3.0, *p* = 0.08).

### Individual Differences in Negative Trauma Appraisals

Negative appraisal scores were similar for boys and girls (means of 6.1 and 6.6, respectively), *t*(412) = 0.69, *p* = 0.49, and were unrelated to age, *r*(414) = -0.02, *p* = 0.74. Scores were significantly higher when the worst traumatic event was more severe, with *M* = 7.5 (*SD* 6.6) for 296 Criterion A events compared to *M* = 3.6 (*SD* 4.5) for 113 events that did not meet this criterion, *t*(407) = 5.71, *p* < 0.001. Moreover, adolescents whose worst event involved abuse (i.e., experiencing sexual or physical abuse, or witnessing domestic violence; *n* = 50) had higher negative appraisal scores than those reporting other forms of trauma (*n* = 364), with mean scores of 9.6 (*SD* 8.3) versus 5.9 (*SD* 5.8), respectively, *t*(412) = 3.90, *p* < 0.001. Participants who reported multiple (*n* = 289) compared to single (*n* = 125) trauma exposures had significantly higher negative appraisal scores (means of 7.4 versus 3.9, respectively), *t*(412) = 5.48 *p* < 0.001. Negative appraisal score was unrelated to years since the worst event, *r*(358) = -0.05, *p* = 0.38. Table 2 displays descriptive statistics and intercorrelations among the remaining continuous variables. A greater number of traumatic event types (cumulative trauma) was associated with more negative appraisals of the worst event. In relation to current circumstances, negative appraisals were moderately correlated with ongoing adversity and more weakly (and negatively) correlated with social support.

**Table 2:** Descriptive statistics and intercorrelations

Variable					Pearson correlation (*r*)			
		*n*	Mean	(SD)	1.	2.	3.	4.
1.	PTSS	403	15.9	(12.4)	-			
2.	Negative appraisals	414	6.4	(6.3)	0.69	-		
3.	Cumulative trauma	414	2.8	(1.8)	0.40	0.40	-	
4.	Ongoing adversity	413	3.3	(4.1)	0.46	0.44	0.33	-
5.	Social support	414	48.3	(7.1)	-0.23	-0.18	-0.18	-0.30

All correlations significant, *p* < 0.001

To gain greater insight into how these interrelated variables might influence the frequency of negative appraisals, we performed multiple linear regression, with square root-transformed negative appraisal score as outcome measure and gender (dummy-coded *female* = 1), age, cumulative trauma exposure, trauma severity (PTSD Criterion A, dummy-coded *yes* = 1), abusive event (dummy-coded *yes* = 1), ongoing adversity, and social support entered as independent variables. As shown in Table 3, results indicated highly significant effects on negative appraisals for the three trauma measures, as well as for ongoing adversity. Gender, age, and social support, on the other hand, showed no independent associations with negative appraisals. In a final model, the four significant predictors together explained 28.0 % of the variance in negative appraisal scores, *F*(4, 403) *=* 39.2, *p* < 0.001.

**Table 3:** Multiple regression estimates for variables associated with negative trauma appraisals

Independent variable		B	*SE* Β	β
	Gender	0.03	0.12	0.01
	Age	0.02	0.06	0.02
	Criterion A event	0.67	0.14	0.21***
	Abusive event	0.52	0.19	0.12**
	Cumulative trauma	0.19	0.04	0.24***
	Ongoing adversity	0.09	0.02	0.26***
	Social support	0.00	0.01	-0.02

The dependent variable is negative appraisal score (square root-transformed). Gender is coded 0 *male*, 1 *female. N* = 408, **p < 0.01; ***p < 0.001

Given that all trauma characteristics were also associated with PTSS, we investigated whether PTSS might have mediated effects of trauma characteristics on negative appraisals. Addition of PTSS as independent variable in the final regression model described above did result in a significant increase (19 %, *p* < 0.001) in explained variance in negative appraisals. However, with the exception of abusive event (no longer significant, standardized β = 0.06, *p* = 0.08), the main results as presented in Table 3 and summarized above remained highly significant after controlling for PTSS. Results of mediation analyses indicated that the three significant predictors did however have indirect (via PTSS) as well as direct effects on negative appraisals; Criterion A event: direct effect = 0.339, indirect = 0.354, bootstrap *SE* = 0.064, 95 % CI [0.241, 0.498], *p* < 0.001; cumulative trauma: direct effect = 0.079, indirect = 0.107, bootstrap *SE* = 0.025, [0.057, 0.157], *p* < 0.001; ongoing adversity: direct effect = 0.037, indirect = 0.062, bootstrap *SE* = 0.011, [0.040, 0.084], *p* < 0.001.

### Do Negative Appraisals Predict Concurrent Posttraumatic Stress Symptoms?

We hypothesized that adolescents who had the most negative appraisals in relation to their traumatic experiences would have the most severe PTSS. As shown above (see Table 2), measures of negative appraisals, cumulative trauma, ongoing adversity, and social support were significantly intercorrelated with each other, as well as with PTSS. To investigate the association between negative appraisals and symptomatology in greater detail, we therefore conducted hierarchical linear regression analyses.

Because of its established role as predictor of both trauma exposure and PTSS, gender was entered as independent variable in the first step of the regression analysis, followed in the second step by variables related to severity of trauma exposure: whether the worst event met DSM-IV Criterion A, whether it entailed interpersonal violence (abusive event), and the total number of reported traumatic event types (cumulative trauma). The negative appraisal score was added in the third step. In the fourth step, we tested potential effects of ongoing adversity and current perceived social support on PTSS.

Results are summarized in Table 4. The regression analysis identified five variables as significant predictors of PTSS: female gender, cumulative trauma, Criterion A event, negative appraisals, and ongoing adversity; together, these variables explained 49 % of the variance in symptoms. Abusive events, although a significant predictor of negative appraisals, showed no association with PTSS. Participants with high levels of social support tended to have less severe symptoms, but this result did not approach significance. Negative appraisal score was by far the best single predictor of PTSS, explaining 22 % of the variance. Mediation analyses indicated that the effects on PTSS of three of the four significant predictors were partially mediated by negative appraisals (Criterion A event: direct effect = 0.541, indirect = 0.338, bootstrap *SE* = 0.076, 95 % CI [0.207, 0.506], *p* < 0.001; cumulative trauma: direct effect = 0.125, indirect = 0.123, bootstrap *SE* = 0.027, [0.072, 0.181], *p* < 0.001; ongoing adversity: direct effect = 0.055, indirect = 0.068, bootstrap *SE* = 0.013, [0.044, 0.095], *p* < 0.001), although each of these variables also had significant direct effects. Gender, on the other hand, showed only direct effects on PTSS, with no significant mediation by appraisals (direct effect = 0.500, indirect = 0.077, bootstrap *SE* = 0.073, [-0.069, 0.215], *p* = 0.31).

**Table 4:** Hierarchical multiple regression estimates for predictors of current PTSS

Independent variable		B	SE Β	β	ΔR^2^	Adjusted R^2^	Overall F
Step 1					0.025**	0.022	10.00**
	Gender	0.56	0.18	0.16**			
Step 2					0.231***	0.248	33.66***
	Gender	0.61	0.16	0.17***			
	Criterion A event	1.06	0.18	0.27***			
	Abusive event	0.54	0.24	0.10*			
	Cumulative trauma	0.33	0.05	0.34***			
Step 3					0.217***	0.465	70.13***
	Gender	0.50	0.14	0.14***			
	Criterion A event	0.58	0.16	0.15***			
	Abusive event	0.09	0.21	0.02			
	Cumulative trauma	0.15	0.04	0.15***			
	Negative appraisals	0.15	0.01	0.53***			
Step 4					0.014**	0.477	52.80***
	Gender	0.50	0.13	0.14***			
	Criterion A event	0.54	0.15	0.14***			
	Abusive event	0.08	0.21	0.02			
	Cumulative trauma	0.12	0.04	0.12***			
	Negative appraisals	0.14	0.01	0.49***			
	Ongoing adversity	0.05	0.02	0.12**			
	Social support	-0.01	0.01	-0.05			

The dependent variable is PTSD Symptoms (PTSS, square root-transformed). Gender is coded 0 *male*, 1 *female*. The variables age and time since index trauma had no significant effects in preliminary models and were therefore excluded from this analysis. *N* = 403. *p ≤ 0.05; **p ≤ 0.01; ***s ≤ 0.001

Finally, to clarify the association between negative appraisals and clinically significant symptomatology, we examined negative appraisal scores in relation to PTSD diagnoses (full, partial, or no PTSD, as established according to UCLA PTSD-RI criteria). Mean scores for each group showed the expected pattern (full PTSD: *n* = 36, *M* = 16.47, *SD* = 7.9; partial PTSD: *n* = 66, *M* = 8.8, *SD* = 5.5; no PTSD: *n* = 312, *M* = 4.7, *SD* = 5.1). Analysis of variance, with square root-transformed negative appraisal score as dependent variable, confirmed these between-group differences in negative appraisals, *F*(2, 312) = 62.00, *p* < 0.001, and in post hoc comparisons (Games-Howell method), all three groups differed significantly from each other (all *p*s < 0.001).

### Negative Trauma Appraisals as Cause or Consequence of Internalizing Symptoms

To investigate the specificity of the relationship between negative trauma appraisals and PTSS, we repeated the hierarchical linear regression analysis, now with SDQ internalizing symptoms (square root-transformed) as the dependent variable. Independent variables gender and event severity (Criterion A) showed no significant association with internalizing symptoms and were therefore excluded from the model. Results indicated that internalizing symptoms decreased with age (Step 1: standardized β = -0.09, *p* < 0.001). Trauma characteristics were entered in Step 2; both abusive events (β = 0.14, *p* < 0.01) and cumulative trauma (β = 0.28, *p* < 0.001) were associated with greater internalizing symptoms. Post-trauma measures, entered in Step 3, also showed significant effects, with ongoing adversity associated with higher internalizing (β = 0.21, *p* < 0.001) and social support with lower internalizing (β = -0.23, *p* < 0.001). Although there was a significant zero-order correlation between negative appraisal score and internalizing symptoms, *r*(403) = 0.28, *p* < 0.001, addition of appraisals as predictor in Step 4 (β = 0.07, *p* = 0.23) did not improve model fit (ΔR^2^ = 0.003, *p* = 0.23). These results can be interpreted as evidence that negative trauma appraisals were specifically associated with PTSS.

We also considered whether the observed relationship between negative appraisals and PTSS might to some extent be explained by co-occurring internalizing symptoms: that is, adolescents with many internalizing symptoms may have developed more negative trauma appraisals, and the apparent link between appraisals and PTSS might then be spurious. To assess this possibility, all variables with significant effects in the models shown in Table 4 were entered in a new regression model, again with square root-transformed PTSS as dependent variable; SDQ internalizing score was entered as independent variable in a second step. Addition of the internalizing variable did result in slight model improvement, with explained variance in PTSS increasing from 48 to 51 % (*p* < 0.001). Although internalizing symptoms were significantly associated with PTSS in this model (β = 0.19, *p* < 0.001), this resulted in virtually no decrease in the effect of negative appraisal score on PTSS (change in β from 0.50 to 0.49).

## Discussion

The current study assessed negative cognitive appraisals in relation to PTSD symptoms among Sri Lankan adolescents exposed to different types of trauma. More than half of the initial sample of 755 participants reported traumatic experiences; exposure to multiple traumas was common. Due to the design and sampling frame of the study, the 2004 tsunami was the most frequently reported traumatic event, even three years after the disaster. The relatively high prevalence (25 %) of clinically significant PTSD in the trauma-exposed subsample of 414 adolescents included in the current analysis thus reflects, in part, the chronic burden of tsunami-related psychopathology.

Our first goal was to determine which demographic, trauma-related, and post-trauma variables were the most important predictors of negative cognitive appraisals. In line with our expectations, regression analyses confirmed that negative appraisals were higher in adolescents who had experienced greater cumulative trauma, more severe and threatening worst events, and worst events involving interpersonal trauma (physical abuse, sexual abuse, or domestic violence). Furthermore, adolescents who experienced higher levels of ongoing adversity scored higher on the negative appraisal measure. As in previous reports (Bal et al. 2009; Tierens et al. 2012), social support was negatively correlated with appraisals; however, in a regression model including all independent variables, social support was no longer a significant predictor. We found no significant associations between negative appraisal scores and gender, age, or number of years that had elapsed since the worst event. However, it is important to note that the sample had a narrow age range and that the majority of index traumas had occurred when the participants were already in late childhood or early adolescence. Taken together, these findings suggest that characteristics of the traumatic event, lifetime trauma exposure, and current life difficulties are more important risk factors for persistent negative trauma appraisals than demographic variables. This information may prove useful in identifying adolescents with PTSD who could profit most from cognitive interventions.

Our second goal was to clarify the role of negative appraisals in relation to PTSD symptoms. To do so, it was necessary to control for other variables that have been implicated as risk or protective factors. The greater risk of PTSD in girls compared to boys, for example, is well established (Trickey et al. 2012); current findings also confirmed higher PTSS in girls. As expected, more severe trauma (Criterion A), greater cumulative trauma, and ongoing adversity were all associated with more severe PTSS. Although the negative correlation between social support and PTSS initially suggested that social support might buffer the effect of trauma on symptoms, this hypothesis was not confirmed in analyses that controlled for the remaining independent variables. In the full regression model, negative appraisals remained the best single predictor of posttraumatic symptomatology and were also an important mediator in the pathways linking other risk factors to symptoms. Thus, in line with the cognitive model of PTSD (Ehlers and Clark 2000), maladaptive cognitions explained a large proportion of observed individual differences in posttraumatic symptoms. Moreover, negative appraisal scores varied in relation to PTSD diagnostic cut-off scores established for the UCLA PTSD-RI. We conclude that negative trauma appraisals were closely linked to concurrent symptoms, even years after the traumatic experience.

Our third goal was to clarify whether negative appraisals of traumatic events among adolescents were more closely associated with PTSS than with internalizing symptoms. Regression analysis revealed similarities as well as differences in the predictor sets for PTSS and internalizing. Both PTSS and internalizing symptoms were higher when participants had experienced more cumulative trauma and greater ongoing adversity, but other predictors appeared to be more specific. Thus, PTSD symptoms were significantly predicted by female gender and severity of trauma, whereas internalizing symptoms were predicted by younger age, abusive interpersonal trauma, and low social support. With regard to our hypothesis, negative appraisal score - as noted above, the best predictor of PTSS - showed no independent association with internalizing symptoms. While confirming that prior trauma and current stress are correlates of a range of emotional problems in adolescents, these findings point to a more specific role of negative trauma appraisals in relation to chronic PTSS. Further, although the cross-sectional nature of these data does not allow us to rule out the possibility that negative trauma appraisals are simply an epiphenomenon of concurrent posttraumatic stress symptomatology, the current findings indicate that the link between appraisals and PTSS is not an artifact of underlying internalizing symptoms.

The current study has a number of limitations. First, all information was based on adolescent self-reports only. Although children’s reports are preferable to parental reports of children’s symptoms in this age group, the combination of symptom reports from both sources yielded higher PTSD prevalence rates in previous research (Scheeringa et al. 2006) and may thus have been informative in the current study. Clinician-based diagnoses were unavailable. Second, because the study sample was drawn from a tsunami-affected area in a developing country, caution is warranted in generalizing the findings to other populations. Both the types of trauma experienced and cumulative lifetime exposure are likely to vary from one geographic region and culture to another; as the current data strongly suggest, these differences may be reflected in the nature and intensity of negative trauma appraisals. Finally, the cross-sectional design of the main study means that findings refer to concurrent associations, precluding conclusions about causality. To date, prospective studies in children have supported the notion that negative appraisals play an important causal role in the pathway from acute to chronic disorder (Ehlers et al. 2003; Meiser-Stedman et al. 2007; Salmon et al. 2007; Bryant et al. 2007), although studies specifically testing this hypothesis have yielded mixed results (Meiser-Stedman et al. 2009a; Palosaari et al. 2013). Even less is known about the causal role of negative appraisals in the maintenance of established symptoms. Longitudinal studies will be essential to help clarify whether negative appraisals are themselves pathogenic or are simply a correlate of chronic disorder. The scope of our study unfortunately did not allow for a large-scale follow-up.

The current study also has some notable strengths. First, the large sample, with almost full participation in representative schools, increases the reliability and generalizability of the findings. Second, assessment of many different types of trauma (and cumulative trauma) extends the findings of many previous studies that focused on specific traumatic events, such as MVAs or accidental injuries (Bryant et al. 2007; Ehlers et al. 2003). This design also sheds some light on the kinds of traumatic experiences that are most likely to lead to negative cognitions. Third, by assessing internalizing as well as posttraumatic stress symptoms, the study was able to demonstrate a degree of specificity in the relationship between negative trauma appraisals and PTSS. Finally, data collected in a non-Western setting not only replicates earlier findings of the close association between negative appraisals and posttraumatic symptomatology in adolescents, but also extends research on the cognitive model of PTSD in a broader global context.

In closing, we note that the recent revision of PTSD diagnostic criteria in DSM-5 (American Psychiatric Association 2013) makes investigations of negative cognitive processes particularly timely. The fact that “negative beliefs” (D2) and “distorted cognitions and blame” (D3) are among the symptoms listed in the new Cluster D “Negative alterations in cognition and mood” should provide new impetus to understand how such maladaptive cognitions arise in the first place, in addition to increased efforts to develop and apply interventions to reduce these negative thoughts. The heavy global and potentially lifelong burden of trauma-related psychopathology in children and adolescents urges continued research focused on this age group.

## Footnotes

1 Sinhalese translation provided by Dr. F. Neuner
